# The Role of Ca^2+^ Sparks in Force Frequency Relationships in Guinea Pig Ventricular Myocytes

**DOI:** 10.3390/biom12111577

**Published:** 2022-10-27

**Authors:** Roshan Paudel, Mohsin Saleet Jafri, Aman Ullah

**Affiliations:** 1 School of Systems Biology, George Mason University, Fairfax, VA 22030, USA; 2 School of Computer, Mathematical, and Natural Sciences, Morgan State University, Baltimore, MD 21251, USA; 3 Center for Biomedical Engineering and Technology, University of Maryland School of Medicine, Baltimore, MD 20201, USA

**Keywords:** guinea pig, cardiac, ventricular myocyte, ryanodine receptor, force-frequency relation, Ca^2+^ sparks, luminal dependence, adaptation, spark duration, spark amplitude, simulations

## Abstract

Calcium sparks are the elementary Ca^2+^ release events in excitation-contraction coupling that underlie the Ca^2+^ transient. The frequency-dependent contractile force generated by cardiac myocytes depends upon the characteristics of the Ca^2+^ transients. A stochastic computational local control model of a guinea pig ventricular cardiomyocyte was developed, to gain insight into mechanisms of force-frequency relationship (FFR). This required the creation of a new three-state RyR2 model that reproduced the adaptive behavior of RyR2, in which the RyR2 channels transition into a different state when exposed to prolonged elevated subspace [Ca^2+^]. The model simulations agree with previous experimental and modeling studies on interval-force relations. Unlike previous common pool models, this local control model displayed stable action potential trains at 7 Hz. The duration and the amplitude of the [Ca^2+^]_myo_ transients increase in pacing rates consistent with the experiments. The [Ca^2+^]_myo_ transient reaches its peak value at 4 Hz and decreases afterward, consistent with experimental force-frequency curves. The model predicts, in agreement with previous modeling studies of Jafri and co-workers, diastolic sarcoplasmic reticulum, [Ca^2+^]_sr_, and RyR2 adaptation increase with the increased stimulation frequency, producing rising, rather than falling, amplitude of the myoplasmic [Ca^2+^] transients. However, the local control model also suggests that the reduction of the L-type Ca^2+^ current, with an increase in pacing frequency due to Ca^2+^-dependent inactivation, also plays a role in the negative slope of the FFR. In the simulations, the peak Ca^2+^ transient in the FFR correlated with the highest numbers of SR Ca^2+^ sparks: the larger average amplitudes of those sparks, and the longer duration of the Ca^2+^ sparks.

## 1. Introduction

Excitation-contraction (EC) coupling is the process triggered by the electrical excitation (depolarization) of the sarcolemma, through the Ca^2+^ release from the sarcoplasmic reticulum (SR) to the contraction of the cardiac myocyte [1]. A frequency-dependent change in the cytoplasmic Ca^2+^ transient underlies the force generated by the myocytes [2]. When external Ca^2+^ enters the myocyte and causes the release of intracellular Ca^2+^ from the SR, the elevation of Ca^2+^ regulates the strength of the cardiac contraction. The change in contractile force of the cardiac myocytes at different pacing is termed an interval-force relationship or a force-frequency relationship (FFR). FFR is an essential intrinsic regulatory mechanism in cardiac myocyte contraction, to match the demand for increased blood supply [3,4]. 

The frequency-dependent contractile strength of the heart varies with the species of animal. In mammals, such as humans, rabbits, and guinea pigs, the relationship between cardiac contractile force and stimulation frequency (FFR) has been recorded to have a positive slope under physiological rates, which is known as the Bowditch phenomena, and a negative slope at higher frequencies [3,5,6,7,8]. The FFR is found to be negative in small animals, such as rats, mice, turtles, lizards, snakes, and fish [9,10,11,12,13,14,15]. The negative FFR in humans is suggested to show an adaptation of the heart in rapid pacing [16]. A negative FFR and alterations in EC-coupling are critical features in heart failure [17,18]. The positive FFR is crucial for the adaptation during increased physical activities or exercise [19], because force increases with increasing pacing frequency. Cardiomyocytes of failing human hearts display reversal in the FFR; there is a decrease in the contractile performance at higher rates of stimulation [20]. The mechanisms of the FFR primarily depend upon changes in the intracellular Ca^2+^ transients, as well as some other factors such as SERCA pump activities, Na^+^ and Ca^2+^ exchangers (NCX), and β-adrenergic control [4,21,22,23].

The activities of the SERCA pump and extracellular extrusion of Ca^2+^ determine the availability of intracellular Ca^2+^ during systole. In mammals such as humans, rabbits, and guinea pigs, about 65–80% of the Ca^2+^ transient comes from SR Ca^2+^ release, and the remainder from outside the myocyte via the L-type Ca^2+^ channel. In smaller mammals, such as rats and mice, ~92% of Ca^2+^ in the transient comes from the SR [24,25,26]. To maintain a steady train of Ca^2+^ transients, the Ca^2+^ released SR must be pumped back into the SR by SERCA. With the rapid heartbeat found in small mammals, this must be accomplished more rapidly. It has been suggested that the negative force-frequency relationship is due to diminished SR Ca^2+^ release in the case of rapid pacing [27].

This study explores the Ca^2+^ transient and its role in force-frequency generation and how the Ca^2+^ spark amplitude and frequency contribute to the force-frequency relations. The new model findings build on the understanding gained from the previous modeling studies by Jafri and co-workers [28], in that diastolic sarcoplasmic reticulum (SR) [Ca^2+^]_SR_ and RyR2 adaptation increases with increased stimulation frequency, giving rise to the falling amplitude of the myoplasmic [Ca^2+^] transients. A similar conclusion is also made from spark analysis; with the increase of SR Ca^2+^ ([Ca^2+^]_SR_), the mean as well as peak amplitudes of the Ca^2+^ sparks raise the force generated by the heart. With the increased adaptation of RyR2s, both peak and mean amplitudes start falling, giving the frequency-dependent decline in the force generation. 

## 2. Materials and Methods

### 2.1. Computational Model Development

The novel stochastic model of guinea pig cardiac ventricular myocyte excitation-contraction coupling presented here integrates a modified model of stochastic Ca^2+^dynamics from the rat ventricular myocytes model by Hoang-Trong and co-workers [29,30,31] with the common pool model for the Jafri-Rice-Winslow guinea pig ventricular myocyte [28]. The resulting model is a local control, Monte Carlo simulation model, which uses 20,000 stochastically gated Ca^2+^ release units that open in the dyadic subspaces of cytoplasm. The Ca^2+^ release units consist of a cluster of 12 L-type and 49 RyR2 channels, coupled with a dyadic subspace with RyR2 adaptation integrated into the gating mechanism. The Jafri-Rice-Winslow model combined ionic current formulations developed in Luo-Rudy models with a novel formulation of the Ca^2+^ dynamics [28,32,33,34]. Appendix A describes the equations and parameters.

### 2.2. RyR Model

**Figure 1:**
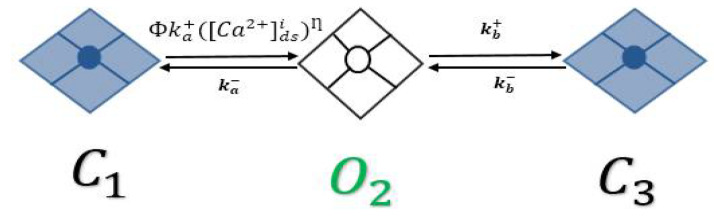
Three-state RyR2 model that produces experimentally constrained Ca^2+^ dependence and adaptation.

In 1998, Jafri et al. [28] developed a model by integrating the L-R II [34] model with a more realistic formulation of the myocyte Ca^2+^ dynamics, by replacing the Ca^2+^ SR release mechanism in Luo-Rudy II with a dynamic RyR2 model with adaptation interacting the L-type Ca^2+^ channels in the dyadic subspace. The RyR2 model had four states: two closed states and two open states. Combining features of that model with the stochastic spark model [29] led to the development of a new three-state model: two closed states and one open state, as shown in Figure 1. The second closed state (C_3_) is an adaptive state. 

In the resting phase, almost all RyR2s stay in the closed state (C_1_); with the elevation of Ca^2+^ in the dyadic subspace, the channels activate into an open state (O_2_) and, after some time, the channels might transition to an adapted state (C_3_). In this model, luminal regulation function (Φ), which depends on SR load ([Ca^2+^]_SR_), modifies the channel opening rate. Therefore, the [Ca^2+^]_SR_ available to be released plays a major role in the developing force-frequency relationship [24]. The release of Ca^2+^ from the SR is calculated by the equation:

(1) $$SR_{rel}=v_{1}\left(N_{O}^{i}\right)\left([Ca^{2+}{]}_{jsr}-[Ca^{2+}{]}_{ds}\right)$$

where $v_{1}$ is the maximal RyR2 single channel Ca^2+^ release rate, [*Ca*^2+^]*_ds_*_,_ Ca^2+^ concentration in dyadic subspace, and [*Ca*^2+^]*_jsr_* luminal Ca^2+^ concentration at the junction. $N_{O}^{i}$ is the number of open RyR2 channels at the ith release site. 

### 2.3. L-Type Ca^2+^ Channel Model

**Figure 2:**
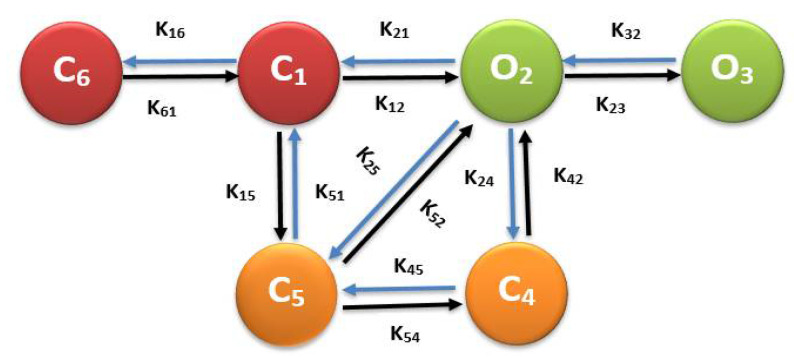
Schematic diagram of the six-state Markov model of the L-type Ca^2+^ channel.

Figure 2 shows the six-state L-type Ca^2+^ channel model used in this work. In this model, states 2 (O_2_) and 3 (O_3_) are open, and states 1 (C_1_) and 6 (C_6_) are closed. C_5_ is the voltage-dependent inactivated state (VDI, O_2_ → C_5_). C_4_, the Ca^2+^-dependent inactivated state (CDI, O_2_ → C_4_), is controlled by the subspace Ca^2+^ in each release site. Increased subspace Ca^2+^ increases the rate of inactivation of LCC and prevents Ca^2+^ overload in the myoplasm by limiting Ca^2+^ entry [35]. The sixth state, C_6,_ was added to the original five-state model of Sun et al. [36], to allow for channels closure during the diastole. The CDI and VDI behaviors were constrained to match the experimental data from Morotti et al. [37]. 

During resting potential, all L-type channels are in a closed state (C_1_, and a change in the membrane potential activate them into an open state (O_2_). A channel in O_2_ state may continue to an open state (O_3_) or transition into the voltage-dependent inactivated state (C_5_), or excess Ca^2+^ in dyadic subspace may bring them into the Ca^2+^-dependent inactivated state (C_4_).

### 2.4. Simulation Frameworks

Simulations using the guinea pig ventricular myocyte model studied Ca^2+^ transients in the cytosol at varying pacing frequencies. Force is assumed to be monotonically related to the size of the cytosolic calcium transient [38]. The foundation of the model was developed in 1 Hz pacing (basic cycle length (~1000 ms). All the parameters were adjusted for 1 Hz and compared all the plots with the experimental results and the previous models work. After this, the model simulated FFR, with basic cycle length as low as 0.2 Hz (1 beat in 5 s) to as high as 8 Hz (8 beats in 1 s). The other simulation frequencies are 0.20 Hz, 0.25 Hz, 0.33 Hz, 0.5 Hz, 1 Hz, 2 Hz, 3 Hz, 4 Hz, 5 Hz, 6 Hz, and 7 Hz. For every pacing frequency, each simulation ran for 10 s to reach a stable train of transients. The simulation data were recorded and plotted against the time. The amplitude and duration of AP, amplitudes of L-type current (I_LCC_), Na^+^-Ca^2+^ current (I_ncx_), Na^+^ current (I_Na_), transient slow outward K^+^ current (I_ktos_), transient outward K^+^ current (I_ktof_), inward rectifier (I_K1_) K^+^ current, cytoplasmic Ca^2+^ concentration ([Ca^2+^]_myo_), NSR Ca^2+^ concentration ([Ca^2+^]_nsr_), and RyR2 opening probability (P_open_) from each beat were measured. Finally, the peak values of each ionic current were collected and plotted against the pacing frequencies. 

The subcellular and molecular analysis of the FFR at Ca^2+^ level was performed by calculating average numbers of Ca^2+^ sparks, Ca^2+^ spark amplitudes, and the Ca^2+^ spark duration. Ten beats were used to compute these data after the simulation arrived in a stable state. After that, an analysis algorithm chose the segments of the systolic phase only and excluded any data from the diastolic phase. From the systolic segment, the dyadic Ca^2+^ transient, which was at least 25 µM tall, was counted as a spark and used to sum the total sparks in that beat. Similarly, the sparks were counted from the rest of the beats, and the average numbers of the sparks were calculated. Likewise, the average amplitudes of the sparks from different beats of the same frequency were measured by adding the amplitude of each beat and dividing the total amplitude by the number of beats. After collecting average spark amplitudes of each beat, the mean amplitude was computed over 10 beats. The average spark duration was calculated using a similar protocol to that used for Ca^2+^ amplitudes. The total spark durations were combined in each beat, the sum was divided by the number of sparks, and the mean spark duration was concluded from the 10 beats.

### 2.5. Numerical Methods

PGI CUDA Fortran was used to compile, execute, and simulate the program on a workstation using the Ubuntu Linux operating system. CUDA (compute unified device architecture) is a parallel computing programming language developed by NVIDIA for graphic processing units (GPUs). The workstations used for the simulations contain Fermi-based C2050 graphics processing cards with CUDA Toolkit 6.0 and higher. To capture calcium dynamics at a single-channel level, a novel computational algorithm Ultra-Fast Markov chain Monte Carlo (UMCMC) method was used for the stochastic gating from the Ca^2+^ release units [39]. Numerical integration of all ordinary differential equations used the explicit Euler method, with an adaptive time step that ranged from 10 ns to 100 ns. 

## 3. Results

### 3.1. Ca^2+^ Transient FFR Curves

**Figure 3:**
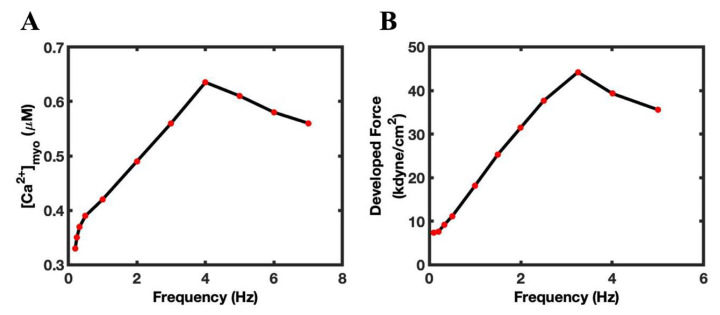
The Ca^2+^ transient FFR curve. (**A**) derived from the model with simulation from 0.2 to 7 Hz, with primary positive FFR (0.2–4 Hz) and secondary phase negative FFR (5–7 Hz). (**B**) An experimental FFR of guinea pig cardiac papillary muscles showed positive FFR (1–3.5 Hz) (data from [40]).

In the simulations, the amplitude of the Ca^2+^ peak transient ([Ca^2+^]_myo_) rose with the increase in the pacing frequency, from 0.20 Hz (0.33 µM) to 4 Hz (0.64 µM), peaking at 4 Hz (Figure 3A). The peak amplitude declines from 5 Hz (0.61 µM) to 7 Hz (0.56 µM). The model showed a positive slope until 4 Hz frequency, and a negative slope after that. Overall, as shown in Figure 3A, the force-frequency relation (FFR) curve was similar to experiments in guinea pig cardiac papillary muscles, as presented in Figure 3B [7,40]. 

**Figure 4:**
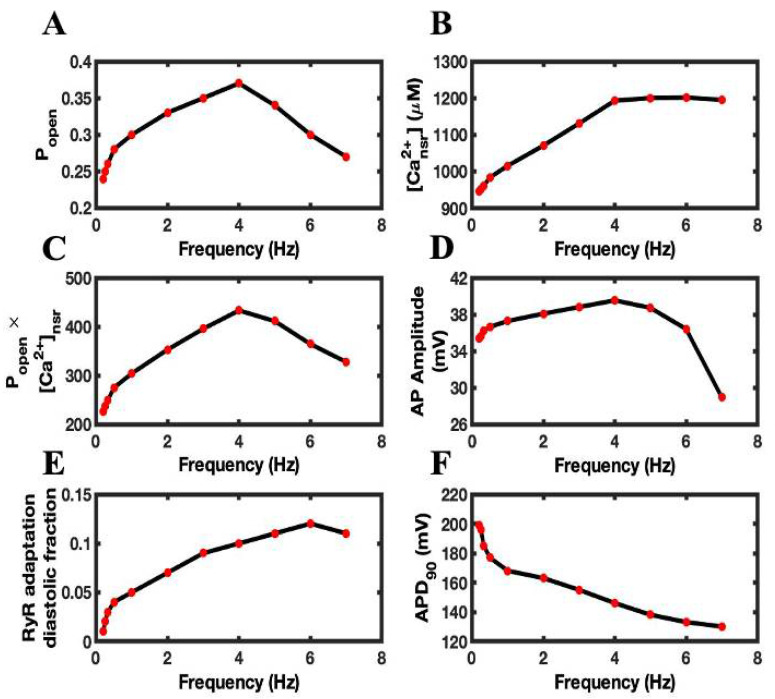
The FFR is determined by Ca^2+^ transient in the intracellular chambers of a myocyte. (**A**) RyR2 open probability (P_open_). (**B**) The network SR Ca^2+^ concentration ([Ca^2+^]_nsr_). (**C**) The peak Ca^2+^ release can be approximated by (P_open_ × [Ca^2+^]_nsr_). (**D**) Action potential amplitude. (**E**) The adaptation of RyR2 during the diastolic phase. (**F**) Action Potential Duration (APD).

With the increase in pacing frequency, the RyR2 open probability (Figure 4A), [Ca^2+^]_nsr_ (Figure 4B), the product of the RyR2 P_open_ with [Ca^2+^]_nsr_ (Figure 4C), and AP amplitude (Figure 4D) behaved similarly to FFR, increasing up to 4 Hz pacing and decreasing thereafter. RyR2 adaptation, on the other hand, increased until 6 Hz and then declined (Figure 4E). When the pacing frequency increases in the model, the APD decreased from 0.2 Hz to 7 Hz (Figure 4F). 

### 3.2. L-Type Current Decreases and I_ncx_ Current Increases with the Rapid Pacing

**Figure 5:**
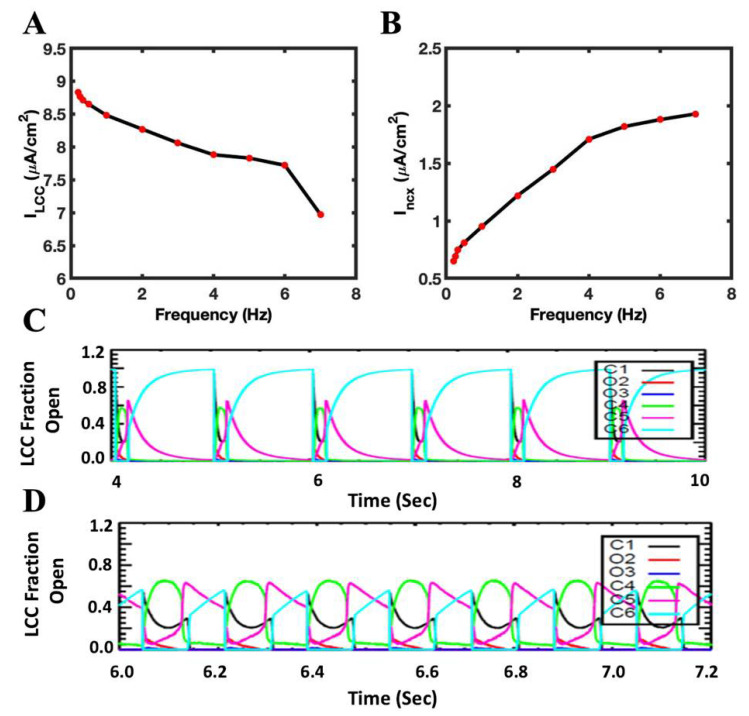
The elevation of cytosolic Ca^2+^ in rapid pacing frequency causes the influx of Ca^2+^ to decrease because of Ca^2+^-dependent inactivation of L-type channels while extrusion of Ca^2+^ increases. (**A**) I_LCC_ amplitude decreases with the increase in the beating rate. (**B**) An increase in I_ncx_ occurs when pacing frequency increases. (**C**,**D**) Different opening, closing, or inactivation states of L-type channels in 1 Hz and 6 Hz pacing frequencies, respectively. C_4_ (green) represents Ca^2+^-dependent inactivated CDI (state), and it is higher in 6 Hz than 1 HZ. C_1_ (black) & C_6_ (cyan) closed states, O_2_ (red) & O_3_ (blue) open states, and C_5_ (magenta) VDI state.

The SR Ca^2+^ concentration rises with the increasing pacing rate, while the Ca^2+^ current in each beat via L-type channel decreases (Figure 5A). On the other hand, the electrogenic I_ncx_ current becomes elevated with the increase in the pacing frequency (Figure 5B). Both these trends are due to the increase in the time-averaged myoplasmic [Ca^2+^]. The Ca^2+^-dependent inactivation of L-type channels is attenuated at lower frequencies (Figure 5C) and rises with the increase in frequency (Figure 5D) (1 Hz vs. 6 Hz). The frequency-dependent increase in RyR2 P_open_ caused an increase in the SR Ca^2+^ release, which in turn caused the LCC channels to become inactive (Figure 5C,D). The loss of L-type current leads to a decrease in the plateau phase in AP, which also decreases the APD with an increasing pacing rate (Figure 4F). The SR Ca^2+^ serves as the feedback mechanism to the L-type channels, and their amplitude decreases with the increase in pacing frequency [41]. In this simulation, the extrusion of Ca^2+^ from Na^+^-Ca^2+^ exchanger constantly increases from 0.2 Hz to 7 Hz (Figure 5B). 

### 3.3. Calcium Sparks Are the Subcellular Mechanisms of FFR 

**Figure 6:**
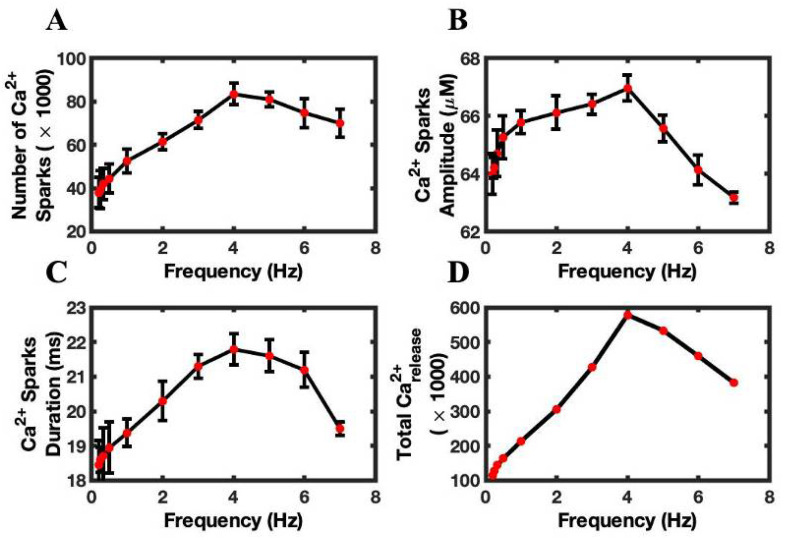
Ca^2+^ spark frequency and amplitudes are better in predicting FFR. (**A**) Ca^2+^ spark frequency. (**B**) Average Ca^2+^ spark amplitude. (**C**) Ca^2+^ spark duration. (**D**) Total Ca^2+^ release approximated with the formula (0.5 × Ca^2+^ spark amplitude × Ca^2+^ spark duration).

The above results demonstrate the role of Ca^2+^ transient in reproducing force-frequency relationship (FFR). The model allows an analysis of how Ca^2+^ spark frequency and Ca^2+^ spark amplitude regulate the Ca^2+^ transient and the resulting contractile force of a myocyte. Ca^2+^ spark properties show similar behavior to Ca^2+^ transients (Figure 3A). The average number of Ca^2+^ sparks in each beat at different frequencies increases from 0.2 Hz to 4 Hz pacing, and the number of sparks gradually decreased thereafter (Figure 6A). The maximum number of sparks appeared at 4 Hz pacing (83,553 ± 5105). The model displayed spark numbers which, when compared to 4 Hz value, decrease by ~5%, ~11%, ~17% with 5, 6, and 7 Hz, respectively. Lukyanenko et al. [42] found that the frequency of the sparks increases with the increase in SR Ca^2+^ load. The Ca^2+^ spark frequency was very high at 4 Hz frequency. In addition to the spark frequency, the average spark amplitudes followed the same trend, with the peak amplitude (62.95 ± 0.55 µM) also occurring at 4 Hz pacing and decreasing at higher pacing frequencies. The SR load also affects the Ca^2+^ spark amplitudes [43]. The model found the largest average amplitudes when SR load was higher at 4 Hz (Figure 6B). A larger amplitude means a greater force would be generated at 4 Hz pacing. The Ca^2+^ spark duration is another activity that showed the same kind of behavior, demonstrating the highest value at 4 Hz (Figure 6C). The model has shown that the total Ca^2+^ release was maximum at 4 Hz (Figure 6D). The total Ca^2+^ release was approximated by multiplying Ca^2+^ spark amplitude and duration and number of sparks and dividing by 2, based on the assumption of a triangular Ca^2+^ flux profile. 

### 3.4. Luminal Dependence and SR Ca^2+^ Play Major Role in FFR

**Figure 7:**
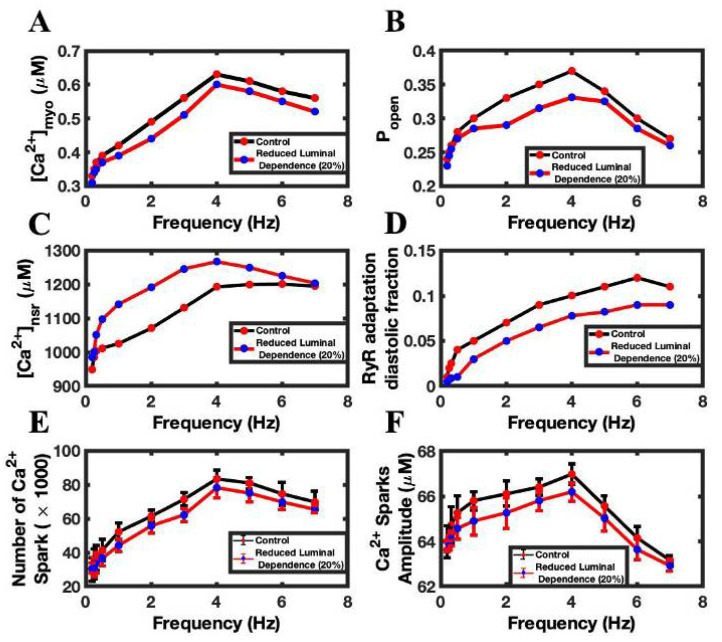
The effect of reduce luminal dependence of RyR2 open probability on frequency-dependent behaviors. (**A**) Peak myoplasmic Ca^2+^ concentration. (**B**) RyR2 open probability. (**C**) Peak NSR Ca^2+^ concentration. (**D**) Diastolic fraction of RyR2 in the adapted state. (**E**) Number of Ca^2+^ sparks. (**F**) Ca^2+^ spark amplitude. Control simulations are shown in black. Simulations with reduced luminal dependence are shown in red.

Luminal Ca^2+^ regulates the RyR2 P_open_ and thereby regulates the dynamics of intracellular Ca^2+^. Higher luminal Ca^2+^ availability increases RyR2 open probability, while a lower value reduces it. Simulations showed that lowering luminal dependency by 20%, in comparison to the control luminal value, reduced the Ca^2+^ transient peaks by ~6% (Figure 7A). The decrease in the luminal dependency lowers the RyR2 P_open_ (Figure 7B) and decreases the amount of Ca^2+^ released from the SR Ca^2+^ via the RyR2, thereby reducing CICR. The consequence of that drop is an increase in the SR Ca^2+^ load, which is shown in Figure 7C. The decreasing luminal activity also played a role in lowering the diastolic fraction of RyR2, because a decrease in the cytosolic Ca^2+^ lowered the RyR2 adaptation rate (Figure 7D), which solely depends upon cytosolic Ca^2+^. Similarly, the numbers of Ca^2+^ sparks (Figure 7E) and the average spark amplitude (Figure 7F) were lower with the reduced luminal Ca^2+,^ because the decreases in SR Ca^2+^ load lowers the RyR2 P_open_ and therefore decreases the number of Ca^2+^ sparks. Simulations showed that ~8% (57,837 ± 18,935 vs. 53,322 ± 17,377) fewer sparks per beat were released with a 20% reduction in luminal dependence than in the control simulation. 

### 3.5. Adaptation Brings Negative Feedback Mechanism to the Open Probability of RyR2

**Figure 8:**
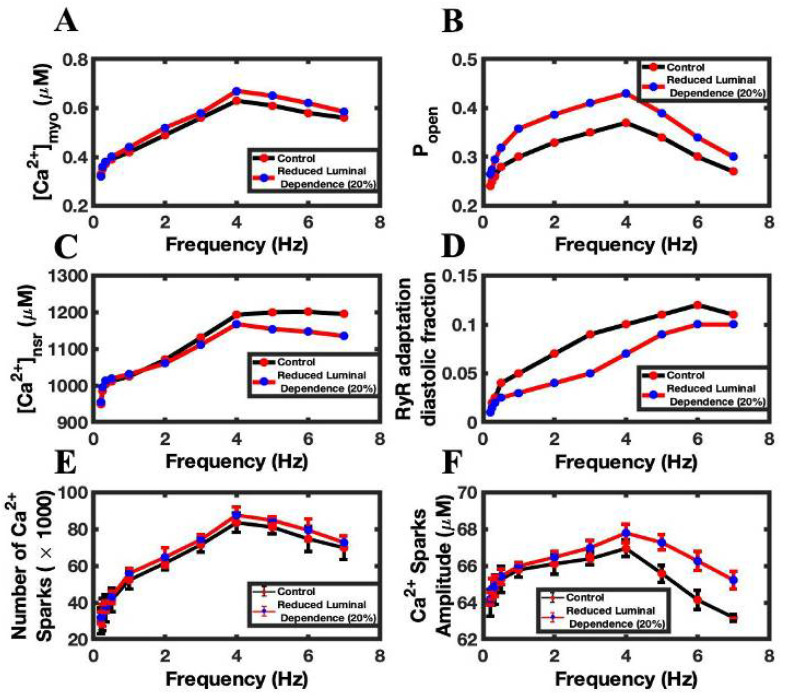
The effects of reduced RyR2 adaptation on frequency-dependent phenomena. (**A**) Peak myoplasmic Ca^2+^ concentration. (**B**) RyR2 open probability. (**C**) Peak NSR Ca^2+^ concentration. (**D**) Diastolic fraction of RyR2 in the adapted state. (**E**) Number of Ca^2+^ sparks. (**F**) Ca^2+^ spark amplitude. Control simulations are shown in black. Simulations with reduced RyR2 adaptation are shown in red.

In the model, adaptation shifts the modal gating behavior of RyR2. A 20% reduction of the adaptation rate of the RyR2 channel is accompanied by a 4% increase in the size of Ca^2+^ transients (Figure 8A), because the RyR2 P_open_ (Figure 8B) went up with the reduction of the RyR2 adaptation. The increased RyR2 P_open_ increase SR Ca^2+^ leak to lower peak diastolic SR Ca^2+^ (Figure 8C) while producing larger Ca^2+^ transients. The RyR2 adaptation fraction decreased as expected (Figure 8D). The increased SR release also produced a greater number of Ca^2+^ sparks (Figure 8E) and larger average spark amplitudes (Figure 8F). In analyzing Ca^2+^ spark behavior, a 5% increase (57,837 ± 18,937 vs. 60,768 ± 19,752) in the Ca^2+^ sparks per beat was observed with this simulation.

### 3.6. The Role of RyR2 Opening Rate Constant in FFR

**Figure 9:**
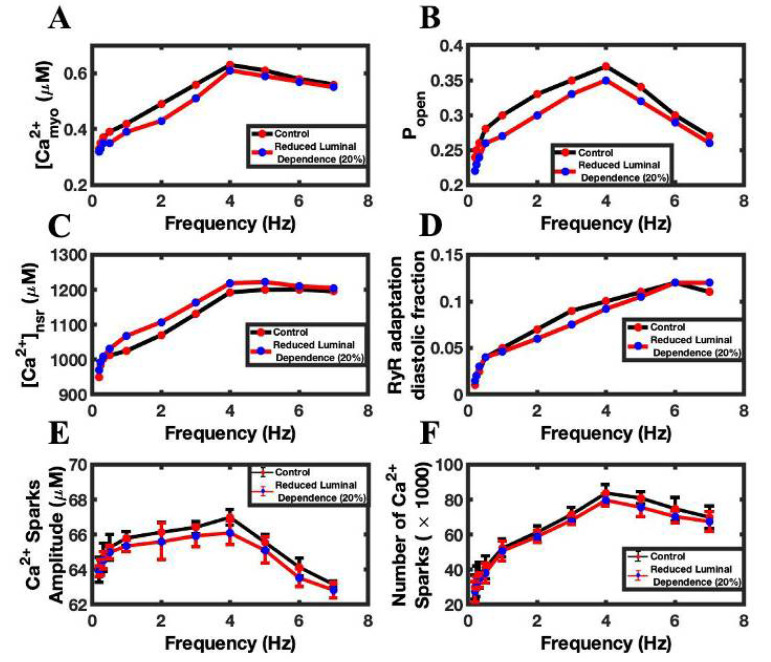
The effects of reducing RyR2 opening rate constant means on frequency-dependent behaviors. (**A**) Peak myoplasmic Ca^2+^ concentration. (**B**) RyR2 open probability. (**C**) Peak NSR Ca^2+^ concentration. (**D**) Diastolic fraction of RyR2 in the adapted state. (**E**) Ca^2+^ spark amplitude. (**F**) Number of Ca^2+^ sparks. Control simulations are shown in black. Simulations with reduced RyR2 activation rate constant are shown in red.

The opening probability plays a major role in the initiation of CICR. A 20% reduction in the RyR2 opening rate constant decreased the peak amplitude of Ca^2+^ transient by 4% (Figure 9A) compared with the control case. The decreased opening rate of RyR2 decreased RyR2 P_open_ (Figure 9B), reducing SR Ca^2+^ leak and thereby increasing diastolic SR load (Figure 9C). The diastolic adaptation of RyR2 (Figure 9D) remains almost the same, because the Ca^2+^ transient did not increase sufficiently when compared to control conditions. The Ca^2+^ sparks (Figure 9E) and spark amplitude (Figure 9F) were also decreased, due to a decrease in RyR2 P_open_. A 3% decrease per beat was found by lowering the RyR2 open constant (57,837 ± 18,937 vs. 55,991 ± 18,182). 

### 3.7. The Role of L-Type Ca^2+^ Channels and NCX in FFR

**Figure 10:**
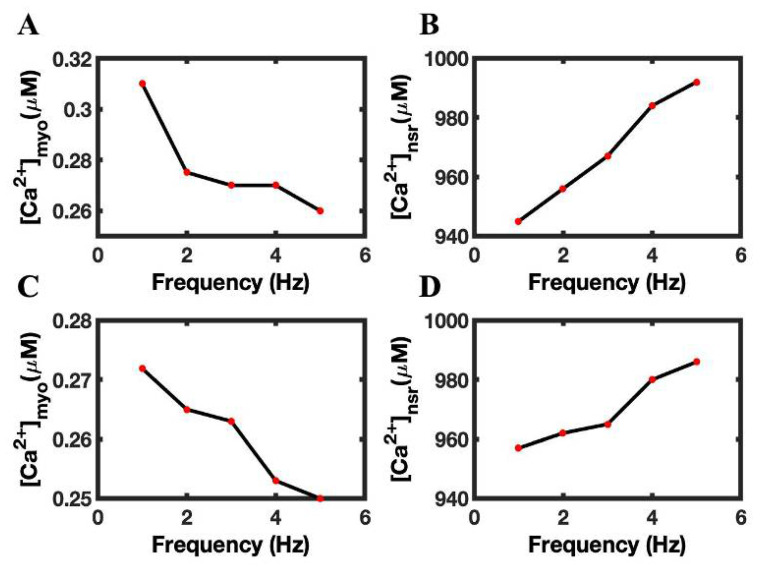
The total myocyte Ca^2+^ content is governed by the balance between efflux via the NCX and influx via the L-type Ca^2+^ channel. A 25% increase in NCX results in negatively sloped FFR and reduced filling of the SR with pacing: (**A**) Peak myoplasmic Ca^2+^ concentration. A 20% reduction in the L-type Ca^2+^ channel current results in a negatively sloped FFR and reduced filling of the SR with pacing: (**B**) Peak NSR Ca^2+^ concentration. (**C**) Peak myoplasmic Ca^2+^ concentration. (**D**) Peak NSR Ca^2+^ concentration.

The previous simulations showed that change properties of the RyR2 could affect FFR. The Ca^2+^ flux through RyR2 play a critical role in SR Ca^2+^ levels, as they regulate the efflux of Ca^2+^ from the SR. On the other hand, the total cellular Ca^2+^ is governed by the transport of Ca^2+^ across the sarcolemma. When the NCX activity is increased by 25%, the FFR has a negative slope (Figure 10A). This occurs because the increase Ca^2+^ efflux across the sarcolemma limits the increase in SR Ca^2+^ with increasing pacing frequency (Figure 10B). In contrast, when Ca^2+^ entry via the L-type Ca^2+^ channels are reduced by 20%, negative FFR is observed in the simulations (Figure 10C). The reduction in Ca^2+^ influx reduced the filling of the SR with increasing pacing frequency, causing the negative FFR (Figure 10D).

### 3.8. Pacing Protocols in FFR

**Figure 11:**
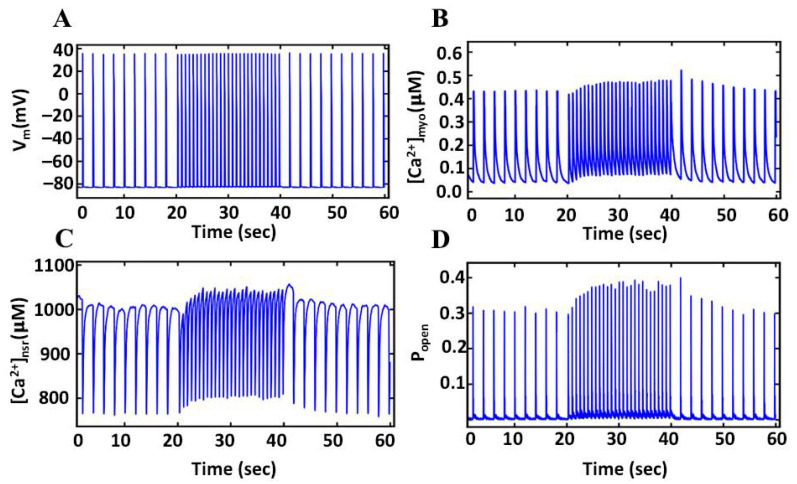
The force-frequency relationship in slow-rapid-slow pacing. (**A**) Membrane potential. (**B**) Myoplasmic Ca^2+^ transients ([Ca^2+^]_myo_). (**C**) NSR Ca^2+^ concentration. (**D**) The peak RyR2 open probability.

Simulations changing the stimulation rate from 0.5 Hz pacing to 1.5 Hz pacing and back to 0.5 Hz pacing are shown in Figure 10. These simulations showed a minimal decrease in the amplitude of AP (39.49 to 39.23 mV) (Figure 11A) from lower to higher and a 9 ms difference in the AP duration (172 ms to 163 ms). Similar to the classic staircase response, the myoplasmic Ca^2+^ transient amplitude increases with a switch to 1.5 Hz pacing rate from 0.5 Hz pacing rate (Figure 11B) and reaches a new steady state due to increased diastolic SR content (Figure 11C). The fraction of RyR2 channels in the adapted state increases with the faster pacing rate, due to the higher Ca^2+^ levels and shorter time for recovery before the subsequent action potential (Figure 11D). On the other hand, when the frequency changes from a 1.5 Hz to 0.5 Hz, there is a transient increase in the Ca^2+^ transient amplitude, followed by a decline to the steady state amplitude. This is due to the recovery for the RyR2 adaptation while the SR load is still elevated during the first set of beats after the rate change.

## 4. Discussion

The frequency-dependent performance of the myocardium changes the contractile strength of the heart. For humans, rabbits or guinea pigs, this force-frequency curve shows biphasic behavior, initially increasing and then decreasing [3,5,6,7,8]. The force generated by the cardiac myocyte is a monotonically increasing function of the amplitude of the myoplasmic Ca^2+^ transient [44]. A newly developed model of the ventricular myocytes of a guinea pig was used to study the role Ca^2+^ dynamics (both Ca^2+^ transients and Ca^2+^ sparks) in shaping the force-frequency curve during the rapidly paced cardiomyocytes. This is an advance of previous work in common pool models, because the newly developed local control model simulated the Ca^2+^ sparks governing excitation-contraction coupling dynamics [28]. The model displayed a positive force-frequency curve, starting from 0.2 Hz and ending at 4 Hz pacing; the calcium transients accompanied by increases to the RyR2 P_O_; RyR2 adaptation; and SR Ca^2+^ load. The RyR2 P_open_ started to decline after 4 Hz pacing. Similarly, the upward trajectory of SR Ca^2+^ ceased and started to decline slowly. However, the fraction of RyR2 channel was observed in the adapted state after 6 Hz. The positive FFR is the characteristic of the mammalian myocardium, including guinea pigs and rabbits [3,45]. Endoh [3] and Varian et al. [46] reported that, in rabbits, the Ca^2+^ transient is positive until 4 Hz pacing and is negative at higher pacing frequencies. The model is used to dissect the molecular mechanism of FFR, as the processes governing the shape of the FFR are only moderately understood [47]. The model demonstrates that the interplay of the RyR2 P_open_ dependence on SR luminal Ca^2+^, SR Ca^2+^ load, and RyR2 adaptation, which govern the shape of the FFR. 

Initially RyR2 adaptation was thought to be relatively slow as a negative control mechanism, but Valdivia et al. [48] found it ~10-fold faster with Mg^2+^ [49]. The adaptation rate for this model was 7 s^−1^. Jafri et al. [28] reported that the adaptation of RyR2 decreased its P_open_ with the beginning of rapid pacing. In this model, the RyR2 adaptation increased as the pacing frequency increased, while RyR2 P_open_ showed a positive slope before 4 Hz and a negative slope above 4 Hz. In the model, we found 5% of RyR2 in adaptation state in 1 Hz; it reached 13% at 6 Hz during diastole, and the number increased during the systolic phase. This RyR2 in the adaptive state played the role in the lesser opening of RyR2 and the release of less SR Ca^2+^ to the cytosol. After running simulations reducing adaptation by 20%, there was an increase in the Ca^2+^ transient amplitude, the number of Ca^2+^ sparks and their amplitudes. The RyR2 P_open_ went up by 7% because both luminal dependency and stimulus by subspace Ca^2+^ provide positive feedback to the P_open_. These results agree with the deterministic model developed by Jafri et al. [28], in that adaptation is important for producing FFR-related behavior. However, the peak of the FFR did not shift with the 20% reduction, suggesting that adaptation is not entirely responsible for the negative FFR. In the whole heart, other mechanisms have been suggested to be important. For example, Puglisi et al. [50] stated that, in the heart, it would not only be RyR2 adaptability, but the heart adapting to generate more force by increasing Ca^2+^ load in the intracellular compartments at a high-frequency rate and increasing myofilament sensitivity [44]. For instance, in β-adrenergic stimulation, it prepares the ventricles to accommodate the higher beating rate. 

It has been reported previously that the faster pacing rate leads to a physiological shortening of APD. Szigligeti et al. [45] changed positive FFR to negative FFR by shortening APD. The model displays this APD restitution with increased pacing frequencies (Figure 4F). In other experiments, as the APD decreased with increased pacing frequency, the maximum rate of ventricular pressure was reciprocal to the APD (Franz, 1983). The frequency dependence of APD is caused by a decrease of the inward current, L-type current, and an increase of outward current, Na^+^-Ca^2+^ exchange current [51]. The model also displayed a frequency-dependent decrease of L-type current (I_LCC_) due to Ca^2+^-dependent inactivation and the increase of I_ncx_ due to activation by Ca^2+^ (Figure 5). The reduction in L-type current means that there are fewer triggering events for Ca^2+^ sparks, which would reduce RyR2 P_open_ as pacing frequency increases. This also contributes to the negative FFR. The local control model captures this mechanism that the previous common pool model could not.

The frequency-dependent increase of SR Ca^2+^ load is the major contributor to positive slope FFR [3]. In guinea pigs, the SR Ca^2+^ load increases with increased pacing rate. However, in rats, a negatively sloped FFR, the SR Ca^2+^ load does not increase significantly with increased pacing frequency [52]. The model displayed increased SR Ca^2+^ load with each pacing rate increase. The higher SR load produced larger Ca^2+^ transients via upward trending RyR2 P_open_-modulated luminal dependency. This role of SR Ca^2+^ has been illustrated previously by the positive staircase phenomenon [17]. An increase in heart rate increases the force of contraction generated by the myocyte; this phenomenon is associated with intracellular Ca^2+^ handling in the myoplasm. In steady-state, with every depolarization, the influx of Ca^2+^ from L-type channels leads to Ca^2+^ release from SR. Myocyte relaxes when Ca^2+^ returns to its original concentration by removing Ca^2+^ from cytosol refill back to the SR by SERCA and efflux via I_ncx_. However, when the pacing frequency increases, the time averaged flux through L-type channels increases because there are more action potentials per second. The increased [Ca^2+^]_myo_ activates SERCA, which increases the sequestration of Ca^2+^ into the SR. The increased SR increases the RyR2 P_open_ in the model, similar to previous experiments [53]. Sobie et al. [54] reported RyR2 activity linearly depends on luminal Ca^2+^; lowering the luminal value shifted the luminal regulation away from the RyR2, and the CICR-related activities were affected and a smaller number of Ca^2+^ sparks, with smaller average amplitudes, were detected. The simulations shown in Figure 10 show that, when Ca^2+^ efflux via the NCX is increased or Ca^2+^ influx via the L-type Ca^2+^ channel is decreased, thereby lowering the total myocyte Ca^2+^, there is limited filling of the SR, with an increase in pacing frequency resulting in a negative FFR.

A positive FFR is an intrinsic contractile property of a ventricular myocyte in mammals, and it is the result of a frequency-dependent acceleration of relaxation [3,5]. The model reproduced this behavior. Varian et al. [46] performed an FFR experiment in rabbits (in vivo) and found similar FFR behaviors as those reported in this article. They have shown that both FFR and Ca^2+^ transient displayed a positive slope until they reached 4 Hz, and the current model followed a similar pattern. They did not report data at higher pacing frequencies, because it was thought that, with the high metabolic demand and greater rundown, data might be compromised. Endoh [3] also reported similar results from rabbit papillary muscle: the Ca^2+^ transient was still positive until 4 Hz, but the contractile force associated with the amplitude of Ca^2+^ transients showed positive FFR from 0.13 Hz to 3.30 Hz, then started to dissociate just before 4 Hz. He also observed negative FFR in rabbits at a higher frequency, and believed this occurred because of altered Ca^2+^ handling and Ca^2+^ overload. The model suggests that the SR loading with increased pacing rate leads to this positive slope of the FFR.

The increase in SR Ca^2+^ load also increases contraction force generated by the myocyte in rapid pacing. The first Ca^2+^ transient from lower to higher pacing becomes shorter, due to smaller recovery time to RyR2 from the previous inactivation, with a shortened diastolic phase. Continuous pacing at increasing frequencies leads to a gradual increase in a positive staircase before reaching a steady state. If the pacing rate decreases from a higher to lower frequency, opposite to the previous condition, the peak of first Ca^2+^ transient becomes greater before gradually a falling to the steady state level. This is because of increased SR load due to an increased influx of Ca^2+^ per unit time in rapid pacing, as well as enough time to reactivate RyR2 channels due to the elongated diastolic phase. We have also found three factors that contribute to a positive staircase, similar to Bers [17], in higher pacing: (a) increased L-type Ca^2+^ current per unit time (not per unit beat); (b) higher diastolic [Ca^2+^]_myo_; and (c) increased SR Ca^2+^ load [Ca^2+^]_sr_ available to be released in subsequent beats. 

The FFR is an important indicator in finding failing or non-failing hearts. The twitch tension (FFR) rises in a normal heart, but does not rise in a failing one [55]. The heart failure is characterized by the decay of contraction and small systolic Ca^2+^ transients [56]. Eisner et al. [56] also explained the small Ca^2+^ transients in two theories: decreased activities of SERCA2a to reload SR and decreased RyR2 P_open._ Although SERCA2a activity was not the direct focus of the current research, with the negative FFR (after 5 Hz), we found decreased SR Ca^2+^ load and reduced RyR2 P_open_, which both played a role in releasing the SR Ca^2+^ in the smaller transients. The model also showed a reduction in I_LCC_ and improved I_ncx_; the former is responsible for decreasing RyR2 activation and the latter competes with SERCA2a to extrude more Ca^2+^ out of the myoplasm. In the model, with the 20% dephosphorylation of the RyR2, we found a small decrease (5%) in the SR Ca^2+^ spark leak, which increased SR Ca^2+^. In the failing heart, the opposite occurs, there is an increase in RyR2 P_open_ due to hyperphosphorylation, but it also increases diastolic Ca^2+^ leak and limits the amount of Ca^2+^ in the SR [57]. In agreement with Endoh [3], the SERCA2a pumps in the failing heart exhaust their capacity to reload SR Ca^2+,^ and the positive FFR turns into negative FFR and the function of the heart is ceased [3]. The model also gave more reasons to believe that decreased SR load caused negative FFR, rather than SR leak. For a normal myocyte, the FFR plot indicates that an increase in pacing rate results in higher Ca^2+^ levels in the myocytes and increases in the contractile force, but a dissociation in that force is necessary to prevent the myocyte from any mechanical damage. A healthy myocardium needs the positive FFR to continue a contractile behavior; in heart failure, the heart loses its ability to refill SR to continue to the frequency-dependent positive FFR [58]. 

RyR2 phosphorylation occurs during beta adrenergic stimulation, which can occur during normal physiology, such as exercise, or during diseases such as heart failure. The phosphorylation of RyR2 increases the diastolic Ca^2+^ leak [57], and a decrease in RyR2 P_open_ should decrease Ca^2+^ leak. In comparing the model data of 1 Hz pacing, we found that the number of Ca^2+^ sparks increase (1441 ± 914) with the reduction in RyR2 opening rate constant. The total diastolic leak rate for 1 Hz pacing was 28,687 ± 1632 for the control simulation, while it was 27,246 ± 1589 for the 20% reduced constant rate simulation. A 20% reduction in the RyR2 channel association rate ($k_{a}^{+}$) for the transition from closed to open states showed that it has a negative effect on RyR2 P_open_; however, in all other simulations, it always remains a fixed value, so it has no role in affecting the outcome. On the other hand, the luminal Ca^2+^ and RyR2 adaptation play a positive and negative role in controlling RyR2 P_open,_ respectively. However, in end-stage heart failure, the expression of SERCA is reduced and the expression of NCX is increased, with the consequence that the SR does not increase its Ca^2+^ content during rapid pacing [59]. In the model, Figure 10 shows how increasing NSR Ca^2+^ efflux abolishes the positive part of the FFR, matching the negative FFR [60] seen in heart failure [3].

The simulations in Figure 11 show that, when there is higher availability of SR Ca^2+^ to be released, the open fraction of RyR2 is also high; conversely, if SR Ca^2+^ load is lesser, the open fraction of RyR2 is also reduced. The interplay of these two performs a major role in the force-frequency relationship, and this is a widely studied phenomenon in both experimental and model settings. This protocol pacing is well studied in both experimental [17] and model settings [28], and the current model was also able to produce similar results. Due to the stochastic opening and closing of Ca^2+^ channels, it was interesting to see the variations in the amplitude of RyR2 open fraction in the consecutive beats, even in a steady state. An increase in the open fraction rate was seen at the beginning of a lower frequency of pacing.

## 5. Conclusions

Simulations with the newly developed model for excitation-contraction coupling in guinea pig ventricular myocyte explored mechanisms behind the experimentally observed force-frequency relations. The continuous refilling of the SR by SERCA pump and enhanced SR Ca^2+^ release is highly critical for increased force-frequency response. The model presented here predicts both the cellular and subcellular mechanism of FFR. In the cellular mechanism, the model suggests, in agreement with previous modeling studies by Jafri and co-workers [28], that diastolic sarcoplasmic reticulum (SR) [Ca^2+^]_SR_ and RyR2 adaptation increases with increased stimulation frequency, giving rise to increasing, rather than falling, amplitude of the myoplasmic [Ca^2+^]_myo_ transients. However, the model also suggests that the reduction of L-type Ca^2+^ current with an increased pacing frequency due to Ca^2+^-dependent inactivation also contributes to the negative part of the FFR. The model was able to give such insight because it also allowed the dissection of these frequency-dependent mechanisms down to the spark level, gaining a deeper perspective into the regulation of cardiac calcium dynamics. 

## Data Availability

Model codes are available as specified above in Supplementary Materials.

## Supplementary Materials

The following supporting information can be downloaded at: https://www.mdpi.com/article/10.3390/biom12111577/s1, Model Code.

## Appendix A

### A.1. Calcium Release

The differential equations describing Ca^2+^ at the release site are shown below, the details can be found in [27,28]

(A1) $$\frac{d{\left[Ca\right]}_{ds}^{\left(i\right)}}{dt}=\frac{\left(J_{ryr}^{\left(i\right)}-J_{efflux}^{\left(i\right)}+(J_{dhpr}^{\left(i\right)}\right)}{\lambda_{ds}}-2\frac{d{\left[CaM\right]}^{\left(i\right)}}{dt}-\frac{d{\left[CaSL\right]}^{\left(i\right)}}{dt}-\frac{d{\left[CaSR\right]}^{\left(i\right)}}{dt}$$

(A2) $$\frac{d{\left[CaM\right]}^{\left(i\right)}}{dt}=k_{CaM}^{+}{\left({\left[Ca\right]}_{ds}^{\left(i\right)}\right)}^{2}\left({\left[CaM\right]}_{T}-{\left[CaM\right]}_{ds}^{i}\right)-k_{CaM}^{-}{\left[CaM\right]}_{ds}^{i}$$

(A3) $$\frac{d{\left[CaSL\right]}^{\left(i\right)}}{dt}=k_{SL}^{+}\left({\left[Ca\right]}_{ds}^{i}\right)\left({\left[SL\right]}_{T}-{\left[CaSL\right]}_{ds}^{i}\right)-k_{SL}^{-}{\left[CaSL\right]}_{ds}^{i}$$

(A4) $$\frac{d{\left[CaSRm\right]}^{\left(i\right)}}{dt}=k_{SRm}^{+}\left({\left[Ca\right]}_{ds}^{i}\right)\left({\left[SRm\right]}_{T}-{\left[CaSRm\right]}_{ds}^{i}\right)-k_{SRm}^{-}{\left[CaSRm\right]}_{ds}^{i}$$

where (*i*) is an index indicating the i^tch^ specific Ca^2+^ release unit, $\lambda_{ds}=V_{ds}/V_{myo}$ is the volume fraction that scale the fluxes, defined based on myoplasmic volume, to the subspace volume compartment. The membrane buffers used here are similar to those used previously [27,28,29].

### A.2. Ryanodine Receptor Type-2 Model

This RyR2 model consists of 20,000 Ca^2+^ release units in each cardiac cell and their state behavior is independent. Each Ca^2+^ release unit contains a cluster of 49 stochastically gated RyR2s and 14 L-type channels. In the model, each Ca^2+^ activated RyR2 is represented by two states–closed and open (see in Figure 1). A transition from a closed state to open state upon Ca^2+^ in dyadic space [Ca^2+^]_ds_ and junctional SR, [Ca^2+^]_jsr_ with $k_{a}^{+}$ as association rate constant and $k_{a}^{-}$. as dissociation rate constant.

The states of the RyR2 are shown in Figure 1 with the transition rates

(A5) $$Ek_{a}^{+}=14{\left({\left[{\mathrm{Ca}}^{2+}\right]}_{ds}^{i}\right)}^{2}\times \left(2.8\times 10^{-4}{\left[{\mathrm{Ca}}^{2+}\right]}_{jsr}^{i}+0.02\right)$$

(A6) $$E{\left[{\mathrm{Ca}}^{2+}\right]}_{ds}^{i}=550$$

Coupling of the RyR2 channels in the cluster uses allosteric coupling energies: $\varepsilon_{OO},\varepsilon_{CC}$ as before [27,28]. There is a possibility that after some time the channels might transition to an adapted state (C3) with the transition rare $k_{b}^{+}$. At each release site, the transition between two cluster states X_i_ and X_j_ is represented as

(A7) $$X_{i}\begin{array}{c} \overset{\chi_{CO}k_{ryr}^{+}N_{C\to O}}{\to }\\ \underset{\chi_{OC}k_{ryrN_{O\to C}}^{-}}{\leftarrow } \end{array}X_{j}$$

where $N_{O\to C}$ and $N_{C\to O}$ represents the number of channels in cluster state X_i_ and X_j_ that can be switched from Open to Closed (Closed to Open). The coupled gating in the cluster is computed by the formula below

(A8) $$\chi_{co}=\exp\left(-{\bar{a}}_{j}\times 0.5\times \left(N_{closed}\varepsilon_{cc}-\left(N_{open}-1\right)\varepsilon_{oo}\right)\right)$$

(A9) $$\chi_{oc}=\exp\left(-{\bar{a}}_{j}\times 0.5\times \left(N_{open}\varepsilon_{oo}-\left(N_{closed}-1\right)\varepsilon_{cc}\right)\right)$$

with ${\bar{a}}_{j}$ is the average allosteric connectivity for each RyR based on mean-field approach [61]. The variables $N_{closed}$ and $N_{open}$ represent the current number of non-conducting, and conducting channels in the cluster state X_i_, respectively.

The non-junctional or ‘rogue’ RyR2 is modelled deterministically using the formula

(A10) $$J_{ryr-nj}=P_{O,ryr-nj}\times \left({\left[{\mathrm{Ca}}^{2+}\right]}_{nsr}-{\left[{\mathrm{Ca}}^{2+}\right]}_{myo}\right)$$

(A11) $$v_{ryr-nj}=v_{ryr}^{T}\times \left[{\mathrm{fraction}}_{\mathrm{nj}-\mathrm{RyR}}\right]$$

(A12) $$\frac{dP_{o,ryr-nj}}{dt}=k_{ryr-nj}^{+}\left(1-P_{o,ryr-nj}\right)-k_{ryr-nj}^{-}P_{0,ryr-nj}$$

$k_{ryr-nj}^{+}$ has the same functional form as $k_{ryr}^{+}$, except the subspace calcium [Ca^2+^]_ds_ in the formula of is now replaced by [Ca^2+^]_myo_. In the model, the fraction of non-junctional RyR2s (ryr-nj) is 5%.

### A.3. L-Type Ca^2+^ Channel Model

The 6-state L-type Ca^2+^ channel (LCC) model, Figure 2, is derived from 5-state LCC model for rat ventricle myocyte from Sun and co-workers with parameters adjusted for the new spark model [35]. Permeation of the L-type Ca^2+^ channel used the Goldman-Hodgkin-Katz (GHK) formalism [60]

(A13) $$I_{dhpr}^{\left(i\right)}=N_{open,\;dhpr}^{\left(i\right)}\frac{P_{dhpr}z_{Ca}^{2}V_{m}}{\frac{RT}{F^{2}}}\frac{{\left[Ca^{2+}\right]}_{ds}^{\left(i\right)}\exp\left(\frac{z_{Ca}V_{m}}{\frac{RT}{F}}\right)-\frac{\beta_{o}}{\beta_{I}\times {\left[Ca^{2+}\right]}_{o}}}{\exp\left(\frac{z_{Ca}V_{m}}{\frac{RT}{F}}\right)-1}$$

with (*i*) represents the index of a release site $N_{open,\;dhpr}^{\left(i\right)}$ is the number of open DHPR channels, ${\left[{\mathrm{Ca}}^{2+}\right]}_{ds}^{\left(i\right)}$ is the calcium concentration in a dyadic subspace), ${\left[{\mathrm{Ca}}^{2+}\right]}_{o}$ is the extracellular calcium concentration; P_dhpr_ is the single channel permeability; Z_Ca_ = 2 is the valence of Ca^2+^ ion; R is the universal gas constant, T is the temperature and F is the Faraday constant. The partition coefficients are $\beta_{0}=0.341,\;\beta_{1}=1$ in the case of Ca^2+^ channel. NOTE: If V_m_ = 0, this formula is used instead

(A14) $$I_{dhpr}^{\left(i\right)}=N_{open,\;dhpr}^{\left(i\right)}P_{dhpr}z_{Ca}F\left({\left[Ca^{2+}\right]}_{ds}^{\left(i\right)}-{\left[Ca^{2+}\right]}_{o}\right)$$

Even though this study did not investigate the role of extracellular calcium, the model also considered the effect of extracellular calcium [Ca^2+^]_o_ on the model by modulating single channel current and shifting the half-activation voltage *V*1/2.

(A15) $$V_{\frac{1}{2}}=-40.344+13.31\times \exp\left(-{\left[Ca\right]}_{o}\times \frac{10^{-3}}{0.053}\right)\;+31.65\times exp\left(-{\left[Ca\right]}_{o}\times \frac{10^{-3}}{11.305}\right)+3$$

In Figure 2, states 2 and 3 are open states; while State 4 is calcium-dependent inactivation and State 5 is voltage-dependent inactivation.

### A.4. Na^+^ Channel Model

The whole-cell Na^+^ current was derived from the formula

(A16) $$I_{Na}=g_{Na}m_{Na}h_{Na}^{3}j_{Na}\left(V_{m}-E_{Na}\right)$$

with the ODE for gating variable follows the first-order differential equation

(A17) $$\frac{dx_{Na}}{dt}=\frac{x_{\infty }-x_{Na}}{\tau_{x}}$$

where *x_Na_* represents the dimensionless gating variables, either *m*, *h*, or *j*, in the range between 0 and 1.0 and $x_{\infty }$ is the steady-state value for the gating variable. The unit for the time constants is in seconds.

(A18) $$h_{\infty }=\frac{1}{1+\exp\left(\frac{V_{m}+76.1}{6.07}\right)}$$

(A19) $$j_{\infty }=h_{\infty }$$

(A20) $$m_{\infty }=\frac{1}{1+\exp\left(\frac{V_{m}+48}{-6.5}\right)}$$

(A21) $$\tau_{m}=\frac{0.00136}{0.32\left(V_{m}+47.13\right)/\left(1-\exp\left(-0.1\left(V_{m}+47.13\right)\right)+0.08\times \exp\left(-\frac{V_{m}}{11}\right)\right)}$$

(A22) $$\mathrm{If}\;V_{m}\geq -0.40\;\tau_{h}=0.0004537\left(1+\exp\left(\frac{V_{m}+10.66}{-11.1}\right)\right)$$

(A23) $$\tau_{j}=\frac{0.01163\left(1+\exp\left(-0.1\left(V_{m+32}\right)\right)\right)}{\exp\left(-2.535\times 10^{-7}V_{m}\right)}$$

(A24) $$\mathrm{If}\;V_{m}<-0.40\;\tau_{h}=\frac{0.00349}{0.81\exp\left(\frac{V_{m}+80}{-6.8}\right)+3.56\exp\left(0.079V_{m}\right)+3.1\times 10^{5}\exp\left(0.35V_{m}\right)}$$

(A25) $$\tau_{j}=\frac{0.00349}{\begin{array}{c} \frac{5\left(V_{m}+37.78\right)}{1+\exp\left(0.311\left(V_{m}+79.23\right)\right)}\left(-12714\exp\left(0.2444V_{m}\right)-3.474\times 10^{-5}\exp\left(-0.04391V_{m}\right)\right)+\frac{0.1212\exp\left(-0.01052V_{m}\right)}{1+\exp\left(-0.1378\left(V_{m}+40.14\right)\right)} \end{array}}$$

(A26) $$h_{\infty }=\frac{0.135\exp\left(\frac{V_{m}+80}{-6.8}\right)}{0.135\exp\left(\frac{V_{m}+80}{-6.8}\right)+3.56\exp\left(0.079V_{m}\right)+3.1\times 10^{5}\exp\left(0.35V_{m}\right)}$$

(A27) $$j_{\infty }=\frac{\frac{V_{m}+37.78}{1+\exp\left(0.311\left(V_{m}+79.23\right)\right)}\left(-12714\exp\left(0.2444V_{m}\right)-3.474\times 10^{-5}\exp\left(-0.04391V_{m}\right)\right)}{\begin{array}{c} \frac{V_{m}+37.78}{1+\exp\left(0.311\left(V_{m}+79.23\right)\right)}\left(-12714\exp\left(0.2444V_{m}\right)-3.474\times 10^{-5}\exp\left(-0.04391V_{m}\right)\right)+\frac{0.1212\exp\left(-0.010527V_{m}\right)}{1+\exp\left(-0.1378\left(V_{m}+40.14\right)\right)} \end{array}}$$

### A.5. K^+^ Channel Models

There are five different K^+^ channel currents (*I_ktof_*, *I_ktos_*, *I_K1_*, *I_Kp_* and *I_Kss_*_)_. The formula for fast and slow transient outward currents (*I_Ktof_*, *I_Ktos_*) respectively are based on the model developed using data observed in mouse [61].

(A28) $$I_{K1}=g_{K1}\times \frac{{\left[K\right]}_{o}}{{\left[K\right]}_{o}+210}\times \frac{\left(V_{m}-E_{K}-1.73\right)}{1+\exp\left(0.0796\left(V_{m}-E_{K-1.73}\right)\right)}$$

(A29) $$I_{kss}=g_{Kss}\times a_{Kss}\times in_{Kss}\left(V_{m}-E_{K}\right)$$

(A30) $$I_{ktof}=\frac{1}{2}g_{Ktof}\times a_{Ktof}3\times in_{Ktof}\left(V_{m}-E_{K}\right)$$

(A31) $$I_{ktos}=\frac{1}{2}g_{Ktos}\times a_{Ktos}3\times in_{Ktos}\left(V_{m}-E_{K}\right)$$

(A32) $$\frac{da_{Kss}}{dt}=10^{3}\frac{\frac{1.0}{1.0+\exp\left(-\frac{V_{m}+22.5}{7.7}\right)}-a_{Kss}}{0.13+0.393\times 0.393\times \exp\left(-0.0862V_{m}\right)}$$

(A33) $$\frac{da_{Ktof}}{dt}=10^{3}\times \left[0.18064\exp\left(\left(V_{m}+30\right)\times 0.3577\right)\times \left(1-a_{Ktof}\right)\;-0.3956\exp\left(-\left(V_{m}+30\right)\times 0.06237\right)\times a_{Ktof}\right]$$

(A34) $$\frac{da_{Ktof}}{dt}=10^{3}\times [\frac{\left(0.000152\exp\left(-\frac{V_{m}+13.5}{7}\right)\right)}{1+0.067083\exp\left(-\frac{\left(V_{m}+33.5\right)}{7}\right)}\times \left(1-a_{ktof}\right)\;-\frac{0.00095\exp\left(\frac{V_{m}+33.5}{7}\right)}{1+0.051335\exp\left(\frac{\left(V_{m}+33.5\right)}{7}\right)}\times i_{Ktof}]$$

(A35) $$\frac{da_{Ktos}}{dt}=10^{3}\frac{\frac{1.0}{1.0+\exp\left(-\frac{V_{m}+22.5}{7.7}\right)}-a_{Ktos}}{2.058+0.493\times \exp\left(-0.0629V_{m}\right)}$$

(A36) $$\frac{di_{Ktos}}{dt}=10^{3}\frac{\frac{1.0}{1.0+\exp\left(\frac{V_{m}+45.20}{5.70}\right)}-i_{Ktos}}{270.0+1050/\left(1+\exp\left(\frac{V_{m}+45.2}{5.70}\right)\right)}$$

(A37) $$I_{Kp}=g_{kp}\left(V_{m}-E_{k}\right)\frac{1}{\left(1.0+\exp(\frac{7.488-v_{m}}{5.98}\right)}$$

with $g_{K1}=0.50;\;g_{Kss}=0.0421;\;g_{Ktof}=0.1598;\;g_{Ktos}=0.00669;\;g_{bK}=0$.

### A.6. Sarcolemmal Pumps/Exchangers

The two extrusion pathways for calcium via SL are plasma-membrane Ca^2+^/ATP-ase (PMCA) and Na^+^/Ca^2+^ exchanger (NCX).

(A38) $$J_{ncx}=\frac{-A_{m}I_{ncx}}{FV_{myo}}$$

with *A_m_* is the SL surface area and V_myo_ is the myoplasmic volume

The NCX is given by the formula

(A39) $$\begin{array}{cc} I_{\frac{Na^{+}}{K^{+}}}= & {\bar{I}}_{\frac{Na^{+}}{K^{+}}}\frac{1}{\left(1+0.01245\exp\left(-0.1\left(V_{m}\frac{F}{RT}\right)\right)\times 0.0365\left(\frac{1}{7}\exp\left(\frac{{\left[Na\right]}_{o}}{67300}\right)-1\right)\exp\left(-V_{m}\frac{F}{RT}\right)\right)}\\ & \times \frac{1}{1+{\left(\frac{21000}{{\left[Na\right]}_{i}}\right)}^{1.5}\frac{{\left[K\right]}_{o}}{{\left[K\right]}_{o}+1500}} \end{array}$$

with ${\bar{I}}_{ncx}$ is the maximal NCX current. The parameters are given in Table A2.

(A40) $$I_{pmca}={\bar{I}}_{pmca}\frac{{\left({\left[Ca^{2+}\right]}_{myo}\right)}^{\eta_{pmca}}}{{\left(K_{m,pmca}\right)}^{\eta_{pmca}}+{\left({\left[Ca^{2+}\right]}_{myo}\right)}^{\eta_{pmca}}}$$

### A.7. Background Currents

We have background currents on the model were computed based upon the Ohm-law

(A41) $$I_{bX}=g_{X}\left(V_{m}-E_{X}\right)$$

Three background currents are calcium, sodium, and potassium

(A42) $$I_{bCa}=g_{bCa}\left(V_{m}-E_{Ca}\right)$$

(A43) $$I_{bNa}=g_{bNa}\left(V_{m}-E_{Na}\right)$$

(A44) $$I_{bK}=g_{bK}\left(V_{m}-E_{K}\right)$$

with the reversal potentials are derived from Nernst equation.

(A45) $$E_{X}=\frac{RT}{Fz_{X}}\ln\left(\frac{{\left[X\right]}_{i}}{{\left[X\right]}_{o}}\right)$$

with *z_x_* is the valance of ion *X*.

### A.8. Non-Specific Ca^2+^-Activated Currents

A set of non-specific currents of Ca^2+^, Na^+^, and K^+^ are computed as below

(A46) $$I_{nsK}=\mathrm{nspK}\times \mathrm{nspCa}$$

(A47) $$I_{nsNa}=\mathrm{nspNa}\times \mathrm{nspCa}$$

(A48) $$I_{nsCa}=I_{nsNa}+I_{nsK}$$

### A.9. Sarcoplasmic Reticulum Ion Pumps

The 2-state reduced model of the Tran-Crampin model [62] was chosen with parameters selected so that the maximum pumping rate per SERXA molecule, is 5 s^−1^.

(A49) $$J_{serca}=2\times v_{serca}$$

(A50) $$v_{serca}=\frac{3.24873\times 10^{12}{\left(K_{myo}\right)}^{2}+K_{myo}\left(9.17846\times 10^{6}-11478.2K_{sr}\right)-0.329904K_{sr}}{D_{cycle}}$$

(A51) $$D_{cycle}=0.104217+17.923K_{sr}+K_{myo}\left(1.75583\times 10^{6}+7.61673K_{sr}\right)\;+{\left(K_{myo}\right)}^{2}\left(6.08463\times 10^{11}+4.50544\times 10^{11}K_{sr}\right)$$

and

(A52) $$K_{myo}={\left(\frac{{\left[Ca\right]}_{myo}}{10^{-3}K_{d,myo}}\right)}^{2};\;K_{sr}={\left(\frac{{\left[Ca\right]}_{nsr}}{10^{-3}K_{d,sr}}\right)}^{2}$$

### A.10. Buffering

The three endogenous buffers of calmodulin (*CaM*), troponin (*Trpn*), and the phospholipids of the SR membrane (*SRm*) are used for the bulk myoplasm. The buffering parameters are listed in Table A3.

(A53) $$\frac{d\left[CaCaM\right]}{dt}=k_{CaM}^{+}\left({\left[Ca\right]}_{myo}^{2}\right)\left({\left[CaM\right]}_{T}-{\left[CaCaM\right]}_{myo}\right)-k_{CaM}^{-}{\left[CaCaM\right]}_{myo}$$

(A54) $$\frac{d\left[CaSRm\right]}{dt}=k_{SRm}^{+}\left({\left[Ca\right]}_{myo}\right)\left({\left[SRm\right]}_{T}-{\left[CaSRm\right]}_{myo}\right)-k_{SRm}^{-}{\left[CaSRm\right]}_{myo}$$

(A55) $$\frac{d\left[CaTrpn\right]}{dt}=k_{Trpn}^{+}\left({\left[Ca\right]}_{myo}\right)\left({\left[Trpn\right]}_{T}-{\left[CaTrpn\right]}_{myo}\right)-k_{Trpn}^{-}{\left[CaTrpn\right]}_{myo}$$

### A.11. Membrane Potential

Our AP model in guinea pig is based upon Luo and Rudy and Jafri and Rice [30,32]. In 1991 Luo Rudy published the first Luo-Rudy Model in ventricular myocytes of guinea pig [31].

During AP, the dynamics of the membrane voltage *V_m_* is governed by the equation

(A56) $$\frac{dV_{m}}{dt}=-\frac{10^{3}}{C_{sc}}\left(\begin{array}{c} \frac{\left(I_{dhpr}^{T}\right)}{A_{m}}+I_{Na}+I_{NCX}+I_{NaK}+I_{pmca}+I_{K1}+I_{Kss}+I_{Ktof}+\\ \;I_{nsCa}+I_{Kp}+I_{Ktos}+I_{background}+I_{app} \end{array}\right)$$

with *NSFU* is the number of Ca^2+^ release sites, and

(A57) $$I_{dhpr}^{T}=\overset{N_{SFU}}{\underset{i=1}{\sum }}I_{dhpr}^{i}$$

(A58) $$I_{background}=I_{bCa}+I_{bNa}+I_{bK}$$

NOTE: The units: membrane potential and reversal potential are in mV, while other parameters are given in the form of unit-density, e.g., the specific membrane capacitance C_sc_ = 1 µF/cm^2^, the ionic currents are given in (µA/cm^2^), except the L-type currents are measured at whole-cell level (µA) which needs to be converted to current density by dividing the membrane surface area A_m_ (cm^2^).

**Table A1:** State Variables and initial conditions.

Variable	Value	Unit
[Ca]_myo_	0.095489	µM
[Ca]_nsr_	1.00891 × 10^3^	µM
[Na]_i_	1.129 × 10^4^	µM
[K]_i_	1.416 × 10^5^	µM
m_Na_	0.0015	Unitless
h_Na_	0.9849	Unitless
j_Na_	0.9905	Unitless
a_Kss_	0.002	Unitless
in_Kss_	10	Unitless
a_Ktof_	0.0021	Unitless
in_Ktof_	1.0	Unitless
a_Ktos_	2.0530697 × 10^−4^	Unitless
in_Ktos_	0.9994	Unitless
$k_{b}^{+}$	5	s^−1^
$k_{b}^{-}$	1	s^−1^

**Table A2:** Parameters used in the model.

Variable	Value	Unit
Faraday constant (F)	9.6485 × 10^4^	C/mol
Universal gas constant (R)	8.314 × 10^3^	mJ/(mol.K)
Temperature (T)	310	K
[Na]o	1.40 × 10^5^	µM
[Ca]o	2.0 × 10^3^	µM
[K]o	5.7 × 10^3^	µM
Cell volume (V_cell_)	36	pL
Myoplasmic volume (V_myo_)	25.84	pL
Network SR volume ($V_{nsr}^{}$)	2.098	pL
Junctional SR volume ($V_{jsr}^{T}$)	1.82 × 10^−1^	pL
Subspace volume ($V_{ds}^{T}$)	3.80 × 10^−1^	pL
Concentration [SERCA]	395	µM
K_d,myo_	880	µM
K_d,sr_	1900	µM
RyR release rate ($v_{ryr}^{T}$)	89.56	s^−1^
Percent nj-RyR	0.05	Unitless
Transfer rate from subspace		
to bulk myoplasm ($v_{efflux}^{T}$)	250	1/sec
Refill rate from nSR		
to jSR ($v_{refill}^{T}$)	2.4	s^−1^
NCX maximum		
current density ($\bar{I_{ncx}}$)	1600	µA/µF
K_d,ncxNa_	8750	µA/µF
K_d,ncxCa_	1380	µA/µF
PMCA maximum		
current density ($\bar{I_{pmca}}$)	0.95	µA/µF
K_d,pmca_	0.5	µA/µF
Na/K maximum		
current density ($\bar{I_{Na/K}}$)	2.0	µA/µF

**Table A3:** Buffering Parameters.

Variable	Value	Unit
[CaM]_T_	24.0	µM
[SRm]_T_	47.0	µM
[Trpn]_T_	140.0	µM
[SL]_T_	200.0	µM
$k_{CaM}^{+}$	2.4	µM^−1^s^−1^
$k_{CaM}^{-}$	380.0	s^−1^
$k_{SRm}^{+}$	115.0	µM^−1^s^−1^
$k_{SRm}^{-}$	100.0	s^−1^
$k_{Trpn}^{+}$	2.37	µM^−1^s^−1^
$k_{Trpn}^{-}$	0.032	s^−1^
$k_{SL}^{+}$	115.0	µM^−1^s^−1^
$k_{SL}^{-}$	1000.0	s^−1^
