# Supplementary material for: The Role of Ca2+ Sparks in Force Frequency Relationships in Guinea Pig Ventricular Myocytes
Source: Biomolecules. 2022 Oct 27;12(11):1577. doi: 10.3390/biom12111577 (PMC9687237; doi:10.3390/biom12111577)
Supplement: Supplementary file 1 [file biomolecules-12-01577-s001.zip › ECC_GPU_FFR/Compartmental_code/src/orderpack-2.0/index.html]

ORDERPACK 2.0 -- Unconditional, Unique, and Partial Ranking, Sorting, and
Permutation Downloadable Fortran 90 source code


## ORDERPACK 2.0 -- Unconditional, Unique, and Partial Ranking, Sorting, and Permutation Downloadable Fortran 90 source code

---

Author: Michel Olagnon

---

## Contents :

:   Introduction

    :   Ranking versus sorting:   Optimization choices:   Programming style:   Examples of use

    :   A word of apology:   Ranking

            :   Unconditional ranking:   Partial ranking:   Unique ranking:   Random permutation: an interesting use of ranking

            :   Sorting

                :   Full sorting:   Partial sorting:   Unique sorting

                :   Download all at once

---

## NEWS

Click here for the latest information about ORDERPACK development and bug correction.

---

## Introduction

The existing fortran code base provides many conventional ranking or sorting routines, but very few specialized ranking or sorting routines.
Specifically, we know of no other fortran code which sorts or ranks only a small proportion of an array (partial ordering).
Such partial ranking routines have applications in statistics for rapidly computing extreme order statistics, finding nearest neighbors, and other clustering operations.
In addition, many applications need to work with only the unique values in an
array (unique ordering).
Such unique ranking routines allow users to isolate individual cases out of a mass of discrete data.
Many times the frequency of the unique values proves interesting (e.g., empirical distributions).
ORDERPACK handles all of these ordering needs.
Also, ORDERPACK contains a partial unique ranking routine.
Such a routine would prove useful in finding a limited number of unique values in an array.
Inversion of orderings becomes difficult when duplicates exist (not a one-to-one relation).
The ORDERPACK inverse ranking routine handles this difficult case.
As an added bonus ORDERPACK provides an unusual routine which allows user controllable partial random permutation of arrays.
ORDERPACK contains conventional or unconditional sorting routines as well.

Finally, many fortran sorting or ranking routines do not take advantage of available memory and cache to maximize performance.
The routines in ORDERPACK have been designed to take advantage of modern machines.

To show the potential speed gains, we conducted an experiment involving 100,000 trials of simulating a random vector of length 500 with duplicates and ranking the 9 smallest unique elements (duplicates discarded).
On a 600 Mhz PIII machine using the CVF 6.1a compiler it took under 2.7 seconds for the unique partial ranking.
In fact, the time was dominated by the simultation of the vector, looping, and other overhead.

A similar experiment involved 100 trials of simulating a random vector of length 1,000,000 and ranking the 20 smallest elements (keeping duplicates).
On a 460 Mhz AlphaStation with Compaq Fortran 90 V5.2, taking care to increase stacksize, partial ranking by itself took 2.3 seconds, *i.e.* 23 milliseconds per vector. In that case, the total overhead for random vector simulation was nearly 1 minute.

Users can freely download ORDERPACK 2.0 from this site.

As time goes by, we hope to extend ORDERPACK, and welcome your suggestions to this aim.

#### Ranking versus sorting

Ranking consists in finding, for each element of a set, its rank in the sorted set, without effectively changing the initial order (or disorder !) of the set.
In many instances, it suits better the actual need of the user, who can then use the index array to order other related sets or to select some elements, than a sorting program would.

Ranking is especially needed when the sizes of the elements are large, and that moving them around is resource-consuming.

#### Optimization choices

We tried to take into account the recent trends in computing to make our compromise choices.
Of course, no two problems are the same, and for some of them, the following decisions may happen to be wrong.
We just hope that for most cases, they will be right.

- Make extensive use of work arrays: Memory can be extended, time cannot.
- Try to reduce the number of operations in the inner loops, even if it increases code size.
- Assume that cache size is relatively small, and try to maximize cache hits.

#### Programming style

Programming style is personal, and difficult to modify when one has been programming for several decades.
Perhaps the following should have been under the *``word of apology''* item: my programming style does not stick tightly to commonly established rules.

If you want to modify my programs, it might be useful to know:

- that I don't state IMPLICIT NONE, I prefer to rely on a good compiler to catch the sort of errors that it catches, and even more of them;
- that I use some naming conventions that go back to the 6-character variable names limit, and the conventions of implicit typing. Most names are made of a first letter indicating the type of the variable, a 3-letter radix defining its fundamental meaning, and one or two suffix letters for qualifiers. I joyfully mix French and English in these names, for instance, IWRKF is an integer index (I) of temporary nature (WRK) representing an upper limit (F -- *Fin* is *End* in French);
- that I used my pretty-printer f90ppr to indent and lay out the source code.

#### Examples of use

In order to make use of Fortran 90 argument passing improvements, it is necessary to make the routine interface known to the calling program.
There are three main ways to implement it:

- Explicit interfaces, either included in the body of the calling routine, or gathered in an ``interface module''. Interfaces for all *default real* ORDERPACK procedures are provided in file interfaces.f90.
  An example of including the interface block in the calling program can be found in the sample program sort7.f90.
- Embedding the routine of interest as a "contained routine" into the calling procedure.
  An example of such way can be found in the follow.f90 program, that rebuilds a curve from a set of X, Y coordinates.
- Embedding the routine of interest into a MODULE, and USEing that module in the procedure that calls the routine.
  This is fine and recommended for small programs, but yet may lead to architecture and *Makefile* problems for large applications.
  Since we wanted to provide generic versions of the routines, and to be F-compatible, this is the way we used here.
  An example of use is provided as the test program tstvalnth.f90.

---

## A word of apology

When one looks at the description of a sorting algorithm, the process seems pretty simple, and can usually hold in 10 to 20 lines of pseudo-code.
But if one wants an optimized program, one takes this simple implementation, and looks for redundant operations, investigates runs with sample data sets with a profiling tool, and is led to duplicate code with slight modifications rather than use tests in inner loops, to process differently the first and the last iterations, or to take into account some special cases that are only special in that they can be done faster.

In the end, the number of lines of source code may be multiplied tenfold, and the readability decreased in a similar proportion.
Unfortunately, this is the price to pay for speed of execution. It was that way when I started programming more than 20 years ago, and I have forsaken any hope that it might become otherwise before I return to dust.
So please accept my apologies that this code is often complex and difficult to read.

---

## Ranking

In some instances, one is not actually interested in modifying the order of the elements in a set, but only in knowing how to access them in increasing -- or decreasing -- order.
Ranking, as it is called, provides the index array I(:) such as the set S(I(:)) is ordered.
One of the advantages of carrying out ranking rather than sorting is that the index array can be computed without the performance penalty of moving the elements around when they are of large sizes.
A similar point is that the index array can be used to index other data.

- #### Unconditional ranking

  Subroutine MRGRNK (XVALT, IMULT)  
  Ranks array XVALT into index array IRNGT, using merge-sort  
  For performance reasons, the first 2 passes are taken out of the standard loop, and use dedicated coding.

  Subroutine MRGREF (XVALT, IRNGT)  
  Ranks array XVALT into index array IRNGT, using merge-sort  
  This version is not optimized for performance, and is thus not as difficult to read as the previous one.
- #### Partial ranking

  Subroutine RNKPAR (XVALT, IRNGT, NORD)  
  Ranks partially XVALT by IRNGT, up to order NORD (refined for speed)  
  This routine uses a pivoting strategy such as the one of finding the median based on the quicksort algorithm, but we skew the pivot choice to try to bring it to NORD as fast as possible.
  It uses 2 temporary arrays, one where it stores the indices of the values smaller than the pivot, and the other for the indices of values larger than the pivot that we might still need later on.
  It iterates until it can bring the number of values in ILOWT to exactly NORD, and then uses an insertion sort to rank this set, since it is supposedly small.

  Subroutine REFPAR (XVALT, IRNGT, NORD)  
  Ranks partially XVALT by IRNGT, up to order NORD  
  This version is not optimized for performance, and is thus not as difficult to read as some other ones.
  It uses a pivoting strategy such as the one of finding the median based on the quicksort algorithm.
  It uses a temporary array, where it stores the partially ranked indices of the values.
  It iterates until it can bring the number of values lower than the pivot to exactly NORD, and then uses an insertion sort to rank this set, since it is supposedly small.

  Subroutine RINPAR (XVALT, IRNGT, NORD)  
  Ranks partially XVALT by IRNGT, up to order NORD  
  This version is not optimized for performance, and is thus not as difficult to read as some other ones.
  It uses insertion sort, limiting insertion to the first NORD values.
  It does not use any work array and is faster when NORD is very small (2-5), but worst case behavior (intially inverse sorted) can easily happen.
  In many cases, the refined quicksort method is faster.

  Integer Function INDNTH (XVALT, NORD)  
  Returns the index of the NORDth value of XVALT (in increasing order)  
  This routine uses a pivoting strategy such as the one of finding the median based on the quicksort algorithm, but we skew the pivot choice to try to bring it to NORD as fast as possible.
  It uses 2 temporary arrays, one where it stores the indices of the values smaller than the pivot, and the other for the indices of values larger than the pivot that we might still need later on.
  It iterates until it can bring the number of values in ILOWT to exactly NORD, and then takes out the original index of the maximum value in this set.

  Subroutine INDMED (XVALT, INDM)  
  Returns the index of the median (((Size(XVALT)+1))/2th value) of XVALT  
  This routine uses the recursive procedure described in Knuth, The Art of Computer Programming, vol. 3, 5.3.3 - This procedure is linear in time, and does not require to be able to interpolate in the set as the one used in INDNTH. It also has better worst case behavior than INDNTH, but is about 10% slower in average for random uniformly distributed values.

  **Note that in Orderpack 1.0, this routine was a Function procedure, and is now changed to a Subroutine.**
- #### Unique ranking

  Subroutine UNIRNK (XVALT, IRNGT, NUNI)  
  Ranks an array, removing duplicate entries (uses merge sort).  
  The routine is similar to pure merge-sort ranking, but on the last pass, it discards indices that correspond to duplicate entries.
  For performance reasons, the first 2 passes are taken out of the standard loop, and use dedicated coding.

  Subroutine UNIPAR (XVALT, IRNGT, NORD)  
  Ranks partially XVALT by IRNGT, up to order NORD at most, removing duplicate entries  
  This routine uses a pivoting strategy such as the one of finding the median based on the quicksort algorithm, but we skew the pivot choice to try to bring it to NORD as quickly as possible.
  It uses 2 temporary arrays, one where it stores the indices of the values smaller than the pivot, and the other for the indices of values larger than the pivot that we might still need later on.
  It iterates until it can bring the number of values in ILOWT to exactly NORD, and then uses an insertion sort to rank this set, since it is supposedly small.
  At all times, the NORD first values in ILOWT correspond to distinct values of the input array.

  Subroutine UNIINV (XVALT, IGOEST)  
  Inverse ranking of an array, with removal of duplicate entries  
  The routine is similar to pure merge-sort ranking, but on the last pass, it sets indices in IGOEST to the rank of the original value in an ordered set with duplicates removed.
  For performance reasons, the first 2 passes are taken out of the standard loop, and use dedicated coding.

  Subroutine MULCNT (XVALT, IMULT)  
  Gives, for each array value, its multiplicity  
  The number of times that a value appears in the array is computed by using inverse ranking, counting for each rank the number of values that ``collide'' to this rank, and returning this sum to the locations in the original set.
  Uses subroutine UNIINV.
- #### Random permutation: an interesting use of ranking

  A variation of the following problem was raised on the internet *sci.math.num-analysis* news group:  
  *Given an array, I would like to find a random permutation of this array that I could control with a ``nearbyness'' parameter so that elements stay close to their initial locations.
  The ``nearbyness'' parameter ranges from 0 to 1, with 0 such that no element moves from its initial location, and 1 such that the permutation is fully random.*

  Subroutine CTRPER (XVALT, PCLS)  
  Permute array XVALT randomly, but leaving elements close to their initial locations  
  The routine takes the 1...size(XVALT) index array as real values, takes a combination of these values and of random values as a perturbation of the index array, and sorts the initial set according to the ranks of these perturbated indices.
  The relative proportion of initial order and random order is 1-PCLS / PCLS, thus when PCLS = 0, there is no change in the order whereas the new order is fully random when PCLS = 1.
  Uses subroutine MRGRNK.

  The above solution found another application when I was asked the following question:  
  *I am given two arrays, representing parents' incomes and their children's incomes, but I do not know which parents correspond to which children.
  I know from an independent source the value of the correlation coefficient between the incomes of the parents and of their children.
  I would like to pair the elements of these arrays so that the given correlation coefficient is attained, i.e. to reconstruct a realistic dataset, though very likely not to be the true one.*

  Program GIVCOR  
  Given two arrays of equal length of unordered values, find a "matching value" in the second array for each value in the first so that the global correlation coefficient reaches exactly a given target  
  The routine first sorts the two arrays, so as to get the match of maximum possible correlation.
  It then iterates, applying the random permutation algorithm of controlled disorder ctrper to the second array.
  When the resulting correlation goes beyond (lower than) the target correlation, one steps back and reduces the disorder parameter of the permutation.
  When the resulting correlation lies between the current one and the target, one replaces the array with the newly permuted one.
  When the resulting correlation increases from the current value, one increases the disorder parameter.
  That way, the target correlation is approached from above, by a controlled increase in randomness.
  Since full randomness leads to zero correlation, the iterations meet the desired coefficient at some point.
  It may be noted that there could be some cases when one would get stuck in a sort of local minimum, where local perturbations cannot further reduce the correlation and where global ones lead to overpass the target.
  It seems easier to restart the program with a different seed when this occurs than to design an avoidance scheme.
  Also, should a negative correlation be desired, the program should be modified to start with one array in reverse order with respect to the other, i.e. coorelation as close to -1 as possible.

---

## Sorting

- #### Full sorting

  Subroutine INSSOR (XVALT)  
  Sorts XVALT into increasing order (Insertion sort)  
  This subroutine uses insertion sort.
  It does not use any work array and is faster when XVALT is of very small size
  (< 20), or already almost sorted, but worst case behavior (intially inverse sorted) can easily happen.
  In most cases, the quicksort or merge sort method is faster.

  Subroutine REFSOR (XVALT)  
  Sorts XVALT into increasing order (Quick sort)  
  This version is not optimized for performance, and is thus not as difficult to read as some other ones.
  This subroutine uses quicksort in a recursive implementation, and insertion sort for the last steps with small subsets.
  It does not use any work array
- #### Partial sorting

  Subroutine INSPAR (XVALT, NORD)  
  Sorts partially XVALT, bringing the NORD lowest values at the begining of the array.  
  This subroutine uses insertion sort, limiting insertion to the first NORD values.
  It does not use any work array and is faster when NORD is very small (2-5), but worst case behavior can happen fairly probably (initially inverse sorted).
  In many cases, the refined quicksort method is faster.

  Function FNDNTH (XVALT, NORD)  
  Finds out and returns the NORDth value in XVALT (ascending order)  
  This subroutine uses insertion sort, limiting insertion to the first NORD values, and even less when one can know that the value that is considered will not be the NORDth.
  It uses only a work array of size NORD and is faster when NORD is very small (2-5), but worst case behavior can happen fairly probably (initially inverse sorted).
  In many cases, the refined quicksort method implemented by VALNTH / INDNTH is faster, though much more difficult to read and understand.

  Function VALNTH (XVALT, NORD)  
  Finds out and returns the NORDth value in XVALT (ascending order)  
  This subroutine simply calls INDNTH.

  Function VALMED (XVALT)  
  Finds out and returns the median (((Size(XVALT)+1))/2th value) of XVALT  
  This routine uses the recursive procedure described in Knuth, The Art of Computer Programming, vol. 3, 5.3.3 - This procedure is linear in time, and does not require to be able to interpolate in the set as the one used in VALNTH/INDNTH. It also has better worst case behavior than VALNTH/INDNTH, and is about 20% faster in average for random uniformly distributed values.
- #### Unique sorting

  Subroutine UNISTA (XVALT, NUNI)  
  Removes duplicates from an array  
  This subroutine uses merge sort unique inverse ranking.
  It leaves in the initial set only those entries that are unique, packing the array, and leaving the order of the retained values unchanged.

---

## Download all at once

- To download all the routines in a single source file, CLICK HERE
- To download all the routines as a tarred gzipped file, CLICK HERE
- To download all the routines as a winzipped file, CLICK HERE

---

Last updated: 2002/01/18

Back to top

---


Michel Olagnon IFREMER Brest / Michel.Olagnon@ifremer.fr
