# Supplementary material for: The Role of Ca2+ Sparks in Force Frequency Relationships in Guinea Pig Ventricular Myocytes
Source: Biomolecules. 2022 Oct 27;12(11):1577. doi: 10.3390/biom12111577 (PMC9687237; doi:10.3390/biom12111577)
Supplement: Supplementary file 1 [file biomolecules-12-01577-s001.zip › ECC_GPU_FFR/Compartmental_code/src/orderpack-2.0/news.html]

ORDERPACK 1.0 -- Unconditional, Unique, and Partial Ranking, Sorting, and
Permutation Downloadable Fortran 90 source code


## ORDERPACK -- Recent news

---

Author: Michel Olagnon

---

## Contents :

:   Index of cited routines

    :   INDMED:   INDNTH:   FNDNTH:   UNISTA:   VALNTH:   VALNTH:   VALMED:   VALMED

    :   On-going at present

        :   Performance improvements:   INDMED:   VALMED:   Development:   UNISTA:   VALMED:   VALNTH (1)

        :   Recent changes

            :   INDNTH/VALNTH bug correction (2001/01/26)

---

- On-going at present
  - Performance improvements:

    I am working on the median and index of median searching routines VALMED and INDMED.

    As of 2001/02/01, I have been conveying the same improvement as below in INDMED, but at present, the performance gain is not as impressive, and it might be faster to use VALMED and look for the index of a value equal to the identified median than to use INDMED.
    My estimate is that it would save about 10% execution time.

    As of 2001/01/27, I have been able to reduce time in VALMED by about 40% through a much better way to sort subsets of 7 elements, as required by the first stage of the algorithm.
    I am in the process of implementing the improvement in INDMED, but at present, the performance gain is not as impressive, and it might be faster to use VALMED and look for the index of a value equal to the identified median than to use INDMED.
  - Development:

    I am working on the next release of ORDERPACK, where we will have routines embedded in F-compatible modules, with a generic name and a specific version for each of the data types Integer, Real, Double precision.

    As of 2001/12/01, only a few routines are still not ready (FNDNTH, VALNTH, VALMED and UNISTA), and I will wait a little more before I put them on the site, so as to make it the upgrade to version 2.0.
    Those who would want an early version should send me an e-mail.
  - Usage help:

    I intend to provide two example programs: the test program for the algorithm to sort subsets of 7 elements, and the F-compatible timing program that I used for the preparation of the first generic modules of the next release of ORDERPACK.

    As of 2001/01/27, this is expected to come during the next weeks.

---

- Recent changes
  - INDNTH/VALNTH bug correction:  

    On 2001/01/26, I realized a line had been forgotten in the correction of the bug below, so it worked only half of the time. If you use INDNTH or VALNTH, please upgrade your version.  
    On 2000/11/04, I found a bug in these routines. Under some circumstances, when there are identical values in the set to be examined, the program would go into an infinite loop.
    A correction was made.

---

Last updated: 2001/02/01

Back to top

---


Michel Olagnon IFREMER Brest / Michel.Olagnon@ifremer.fr
